# Metabolite Profiling Reveals Developmental Inequalities in Pinot Noir Berry Tissues Late in Ripening

**DOI:** 10.3389/fpls.2017.01108

**Published:** 2017-06-30

**Authors:** Amanda M. Vondras, Mauro Commisso, Flavia Guzzo, Laurent G. Deluc

**Affiliations:** ^1^Deluc Laboratory, Department of Horticulture, Oregon State University, CorvallisOR, United States; ^2^Guzzo Laboratory, Department of Biotechnology, University of VeronaVerona, Italy

**Keywords:** uneven ripening, crop heterogeneity, metabolomics, HPLC-MS, fruit composition, *Vitis vinifera*

## Abstract

Uneven ripening in *Vitis vinifera* is increasingly recognized as a phenomenon of interest, with substantial implications for fruit and wine composition and quality. This study sought to determine whether variation late in ripening (∼Modified Eichhorn-Lorenz stage 39) was associated with developmental differences that were observable as fruits within a cluster initiated ripening (véraison). Four developmentally distinct ripening classes of berries were tagged at cluster véraison, sampled at three times late in ripening, and subjected to untargeted HPLC-MS to measure variation in amino acids, sugars, organic acids, and phenolic metabolites in skin, pulp, and seed tissues separately. Variability was described using predominantly two strategies. In the first, multivariate analysis (Orthogonal Projections to Latent Structures-Discriminant Analysis, OPLS-DA) was used to determine whether fruits were still distinguishable per their developmental position at véraison and to identify which metabolites accounted for these distinctions. The same technique was used to assess changes in each tissue over time. In a second strategy and for each annotated metabolite, the variance across the ripening classes at each time point was measured to show whether intra-cluster variance (ICV) was growing, shrinking, or constant over the period observed. Indeed, berries could be segregated by OPLS-DA late in ripening based on their developmental position at véraison, though the four ripening classes were aggregated into two larger ripening groups. Further, not all tissues were dynamic over the period examined. Although pulp tissues could be segregated by time sampled, this was not true for seed and only moderately so for skin. Ripening group differences in seed and skin, rather than the time fruit was sampled, were better able to define berries. Metabolites also experienced significant reductions in ICV between single pairs of time points, but never across the entire experiment. Metabolites often exhibited a combination of ICV expansion, contraction and persistence. Finally, we observed significant differences in the abundance of some metabolites between ripening classes that suggest the berries that initiated ripening first remained developmentally ahead of the lagging fruit even late in the ripening phase. This presents a challenge to producers who would seek to harvest at uniformity or at a predefined level of variation.

## Introduction

That producers seek to define and pursue optimal levels of enologically important metabolites in grapes is understood. However, intra-cluster variation is an important consideration as well, given the link between fruit uniformity and crop quality (Carroll et al., 1978; Selvaraj et al., 1995; Barbagallo et al., 2011; Kontoudakis et al., 2011; Liu et al., 2016). This contrasts the prevalent paradigm wherein the mean amount of a metabolite for a population of berries, rather than the variability inherent to that population, influences harvesting decisions. Whether optimal levels of traditional markers that influence harvest decisions (sugars, pigments, tannins, and organic acids) coincide with desirable levels of heterogeneity is largely unexplored.

Several studies have examined the ways in which fruits within a cluster vary, why fruits may initiate ripening unevenly, and means of managing heterogeneity (Cawthon and Morris, 1982a,b; Coombe, 1992; Fernandez et al., 2006; Friend et al., 2009; Gray and Coombe, 2009; Pagay and Cheng, 2010; Calderon-Orellana et al., 2014b; Gouthu and Deluc, 2015). The uneven onset of ripening in a cluster (véraison) has been attributed to fruits’ seed content, weakly to flowering time, and the interplay of hormones (Böttcher et al., 2010; Gouthu and Deluc, 2015; Vondras et al., 2016). Then, between véraison and harvest, intra-cluster variance (ICV) is reduced in terms of gene expression, °Brix, color index, and size (Gray and Coombe, 2009; Pagay and Cheng, 2010; Gouthu et al., 2014). However, differences at harvest are still observed and not without consequences. Although one study found no significant relationship between crop price and crop heterogeneity (Calderon-Orellana et al., 2014a), Carroll et al. (1978) showed that wines from fruits belonging to the least and most advanced berries had the lowest sensory scores. They observed differences in sugar, pH, titratable acidity, wine tannins and color between different classes of berries. In Syrah, larger berries at commercial harvest had lower quality characteristics and a yellow–green color indicative of incomplete maturity and possibly higher seed catechin extractability (Barbagallo et al., 2011). In recognizing that substantial variation at harvest limits accurate determination of phenolic maturity, Kontoudakis et al. (2011) also showed that wines from higher density (high sugar) berries were associated with higher ethanol content, pH, color intensity, total phenolic indexes, anthocyanins, and polymerization of proanthocyanidins and lower titratable acidity and bitterness; the resulting wines were higher quality and better balanced. In another study, less-dense grapes contributed fewer anthocyanins and more seed tannins than skin tannins, detrimentally affecting wine composition, while denser berries had the highest total phenolic content (Liu et al., 2016). Whether or not this variability is predominantly due to developmental differences is unexplored, though previous reports have demonstrated variation associated with other factors, like fruit position within clusters (Kasimatis et al., 1975; Tarter and Keuter, 2005; Pagay and Cheng, 2010; Pisciotta et al., 2013).

If fruits are developmentally equals, then dynamic tissues should undergo key developmental transitions, like véraison and dehydration, uniformly. Therefore, perhaps the most appropriate time to make such assessments is as those transitions occur. Late ripening, which we define here as the period of extended ripening immediately following ripeness (Coombe, 1995), is a period during which fruits dehydrate. Distinctive wines are produced using both on- (Rolle et al., 2009; Bowen and Reynolds, 2015; Khairallah et al., 2016; Lukić et al., 2016) and off-vine (Bellincontro et al., 2004; Costantini et al., 2006; Moreno et al., 2008; Toffali et al., 2011; Zenoni et al., 2016) dehydration strategies (Figueiredo-González et al., 2013). Both practices have similar effects on sugars, secondary metabolism, and cell integrity (Zamboni et al., 2008) and desiccation can produce responses analogous to those of water stress (Bellincontro et al., 2009). This developmental window is not only important for winemakers because of the dramatic metabolic changes that occur, but also because it might be used to better appreciate developmental inequality within a cluster.

The purpose of this study was to determine whether intra-cluster variation late in ripening was linked to differences in developmental progress that are observable as fruits unevenly being ripening at véraison. Toward this objective, fruits were tagged as members of qualitative developmental categories or “ripening classes” based on their color at véraison and collected as fruits passed what would be considered commercial harvest into a stage that could be described as on-the-vine withering. If developmental differences persisted between fruits in a cluster, they might be best captured (1) as fruits transition into this stage and (2) in “dynamic” tissues (tissues that demonstrate they are changing within the window observed). A multivariate technique called Orthogonal Projections to Latent Structures-Discriminant Analysis (OPLS-DA) was used to clarify the extent to which different berry tissues remained dynamic in the late ripening period and to determine if and due to which metabolites berries late in ripening could be segregated based on their developmental category at véraison. It was concluded that (1) overall, fruits that were developmentally distinct at véraison remain distinguishable late in ripening, (2) skin, pulp, and seeds were not equally dynamic in the late ripening period, and (3) this period was marked by both reductions and expansions in variation for many metabolites, though most annotated metabolites showed no significant changes in ICV over the period.

## Materials and Methods

### Experimental Design

This study was conducted in 2011 at the Oregon State University Woodhall experimental vineyard in Alpine, Oregon. Pommard grapevines, clones of *Vitis vinifera* L. cv. Pinot noir, grown on 101-14 rootstock, and trained in a double Guyot system with vertical shoot positioning were used. The five vines used for this study were managed using standard viticultural techniques. On each plant, six primary clusters were chosen on both the east (three clusters) and west (three clusters) side of plants.

A non-invasive tagging technique was used to label four qualitatively distinct ripening classes of fruits at véraison (Lund et al., 2008; Gouthu et al., 2014) on September 10th. Here, véraison is defined as when ∼50% of the cluster remains green, while ∼50% has visibly initiated ripening. Among these fruits, Green Hard (GH), Green Soft (GS), Pink (PS), and Red (RS) fruits were randomly selected and tagged throughout each of the selected clusters. GH and GS were completely green with no evidence of color-change. GH and GS were distinguished by touch, with GH having no perceptible deformation. PS often exhibited green and pink marbling or were light pink in color. RS berries were dark pink or red. Within each cluster, representatives of each ripening class were tagged at véraison using different colored strings. Then, six berries from each ripening class were sampled from each plant 34, 41, and 48 days after véraison: October 14th (t1), 21st (t2), and 28th (t3). In this study, one biological replicate is equal to six berries of a particular ripening class and from one of the plants used. Sampled berries were immediately frozen on dry ice and then stored at -80°C. These sampling dates corresponded approximately to stage 39 (overripe) in the modified Eichhorn-Lorenz system for classifying grapevine growth stages (Coombe, 1995).

### Berry Measurements

Total soluble solids and color were measured per berry (*n* = 5). A SPER Scientific digital refractometer (Scottsdale, AZ, United States) was used to measure total soluble solids in units of degrees Brix (°Bx) and a Konica Minolta CR-300 chroma meter (Minolta Corp, Osaka, Japan) was used to quantitatively measure color [lightness (L), hue angle (h), and chroma (C)]. The color index of each berry was calculated as previously described (Carreño et al., 1995) and computed as (180 - h)/(L + C).

### Metabolite Extraction

Approximately 40 mg of lyophilized material were weighed and extracted with 20 and 40 volumes (w/v) of cold, 90% methanol for pulp and seed, respectively. Skin tissues were subjected to 40 volumes (w/v) of cold 89.9% methanol acidified with 0.1% (v/v) of formic acid (Toffali et al., 2011). The extracts were vortexed, sonicated in an ice-filled ultrasonic bath (Falc Instruments, Bergamo, Italy) for 20 min at 40 kHz, kept in darkness for 2h at 4°C and finally centrifuged at 13000 rpm for 10 min at 4°C. Supernatants were collected and stored at -20°C. The 200 μL of each extract were diluted 1:2 with LC-MS-grade water and filtered with Minisart RC 4 membrane filters (0.2 μm diameter pores, Sartorius) prior to injection into the HPLC-MS system.

### Metabolite Separation, Detection, and Annotation

Twenty microliters of each diluted sample were drawn through a 508 Autosampler (Beckman Coulter, Fullerton, CA, United States) system and injected to a Beckman Coulter Gold 127 HPLC system (Beckman Coulter, Fullerton, CA, United States) equipped with a C18 guard column (7.5 mm × 2.1 mm) in front of an Alltima HP C18 column (150 mm× 2.1 mm, particle size 3 μm; Alltech Associates Inc, Derfield, IL, United States). Samples were analyzed randomly and in technical duplicate. The chromatographic solvents, conditions, and gradient are described in Anesi et al. (2015).

Metabolite detection was carried out with a Bruker ion trap Esquire 6000 (Bruker Daltonics GmbH, Bremen, Germany) equipped with an ESI ion source with the following specifications: 10 L/min for N_2_ drying gas and 50 psi for the N_2_ nebulizing gas heated at 350°C. The analyses were performed in negative and positive alternate modality, setting a target mass of 400 m/z and a scan range of 50–3000 *m/z*. Metabolite fragmentation was performed up to MS^3^ by using Helium gas and setting the fragmentation amplitude at 1 V. Chromatographic data were recorded up to 55 min with Esquire Control v5.2 software and the.d generated files were processed with the proprietary Data Analysis v3.2 (Bruker Daltonics), converted in net.cdf files and analyzed with open-source MZmine 2.10^1^ software to create a data matrix reporting feature peak areas. After peak deconvolution, alignment and gap filling performed by MZmine can result in few missing values in the data matrix; such values were considered “missing” and not as zero (Commisso et al., 2017). The subsequent multivariate statistical analyses were carried out with SIMCA v13.0 (Umetrix AB, Umea, Sweden).

Metabolite annotation were made by comparing the *m/z*, retention time and fragmentation pattern (MS/MS and MS^3^) of the detected signals with an in-house library of authentic commercial standards or, in their absence, with data reported in literature or online databases^2^^,^^3^. The confidence of each metabolite annotation was classified as prescribed by Sumner et al. (2007) and is defined in **Data Sheet 1**.

### Statistical Analyses

Several statistical methods were used to explore intra-cluster differences (differences among the ripening classes) and changes within the cluster over time.

Pareto scaling was applied to all analytical methods (van den Berg et al., 2006; Toffali et al., 2011). PCA was used to identify and remove 20 probable outliers from the 180 samples. The data from the remaining 160 samples (*n* = 3–5) were analyzed by PCA and OPLS-DA using SIMCA 13.0 (Umetrix AB, Umea, Sweden) to identify metabolites or biomarkers that accounted for differences between ripening groups and distinguished time points. OPLS-DA models were cross validated by ANOVA (*p*-value < 0.05) and equivalent PLS-DA models were fit and tested by permutation (200 permutations) to avoid overfitting (Triba et al., 2014). In addition to meeting these criteria, only models with high, cross-validated predictability (Q2 > 0.50) were considered as high confidence (Triba et al., 2014; Anesi et al., 2015). All features were used in SIMCA 13 analyses; only annotated metabolites are shown in figures and discussed.

ICV was estimated by averaging the five biological replicates within a ripening class and calculating the variance across the four ripening classes at a single time point for each annotated metabolite. Unidentified features/metabolites were not included in this analysis. An *F*-test for variance was used to test for significant changes in ICV between t1, t2, and t3 for each metabolite. In addition, a Tukey-test was used to test for significant differences in relative metabolite amount between ripening classes.

### Interactive Figures

Figures were constructed using Prism (GraphPad Software Inc., San Diego, CA, United States), SIMCA 13.0, and plot.ly, an online graphing resource. Though still images are presented in this manuscript, the interactive equivalents of plot.ly-generated figures are provided as supplemental HTML files for an enriched exploration of the results (**Presentation 1**). Given the interactive files, readers can include or exclude groups of metabolites by clicking on them in the figure legends, zoom in and out of specified regions of the plots, rotate three-dimensional figures, and identify individual data points which have not been labeled in the still images provided herein. These figures are helpful for visualizing the described trends in the data.

## Results

### Developmental and Metabolic Inequality

We sought to determine whether intra-cluster variation late in ripening was associated with developmental inequalities that were apparent at véraison. This was enabled by tagging developmentally distinct “ripening classes” of fruits at véraison (GH, GS, PS, RS) and then sampling them late in ripening.

**FIGURE 1:**
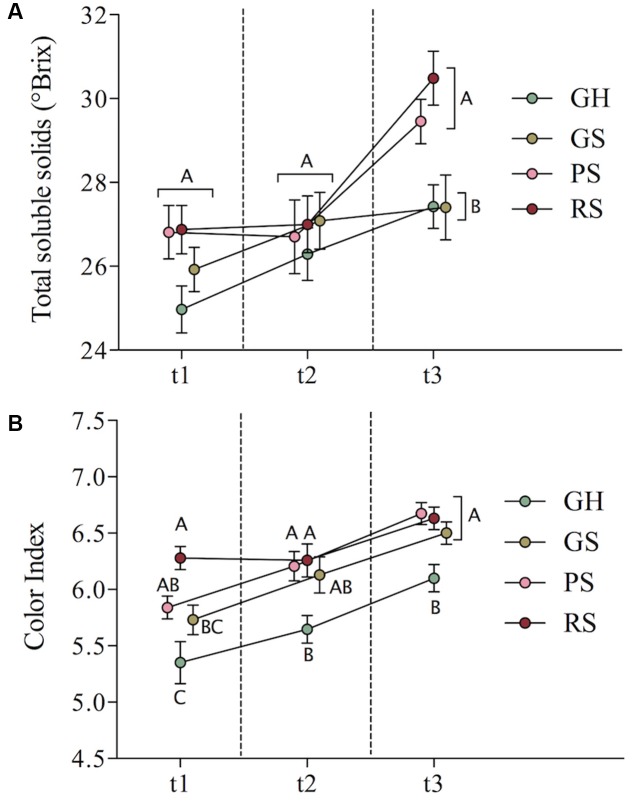
Mean total soluble solids **(A)** and color index **(B)** for the four ripening classes over time with standard error bars shown. Different letters indicate significant differences at a single time point, Tukey HSD-test, *p*-value < 0.05.

In terms of total soluble solids (**Figure 1A**), the four ripening classes trended toward uniformity at t2, but this uniformity was short-lived. Green fruits (GH and GS) had significantly lower °Brix than more advanced fruits (PS and RS) at t3. No significant differences were observed among the ripening classes at t1 or t2 (**Figure 1A**). With respect to berry color, GH showed significantly lower color index than PS and RS at every collection time and lower color index than GS at t2 and t3 (**Figure 1B**). The color index of GS was similar to PS at t1 and indistinguishable from PS and RS at t2 and t3. Together, **Figures 1A,B** examined separately present seemingly distinct narratives concerning fruit development and changes in ICV. ICV as defined by total soluble solids (°Brix) describes clusters trending toward uniformity from t1 to t2, with inequalities reappearing after t2. Alternatively, ICV as defined by color index suggests that at and after harvest, differences between the berries that were the most and least advanced at véraison persist without an obvious point of relatively high uniformity. These initial observations indicate that (1) perception of cluster uniformity depends on the metabolites being measured, (2) developmental inequalities persist, though they may be temporarily masked, and (3) metabolite uniformity does not necessarily suggest developmental uniformity. This also suggests limits to how much developmental inequality observed at véraison is actually mitigated by harvest. Importantly, **Figure 1A** shows that fruits that were relatively advanced at véraison also initiated the dehydration stage first, as by this time increases in sugars are linked to dehydration rather than import.

Untargeted HPLC-MS was used to further assess ICV of metabolites separately in berry seeds, skin, and pulp. Following data acquisition, 139 metabolites were annotated using an in-house library (**Data Sheet 1**). Including all features, annotated and unknown, Orthogonal Projections to Latent Structures – Discriminant Analysis (OPLS-DA) was used to determine whether the ripening classes were distinguishable late in ripening, whether fruits from t1, t2, and t3 were distinguishable overall and if so, which metabolites account for the segregation of different groups.

**FIGURE 2:**
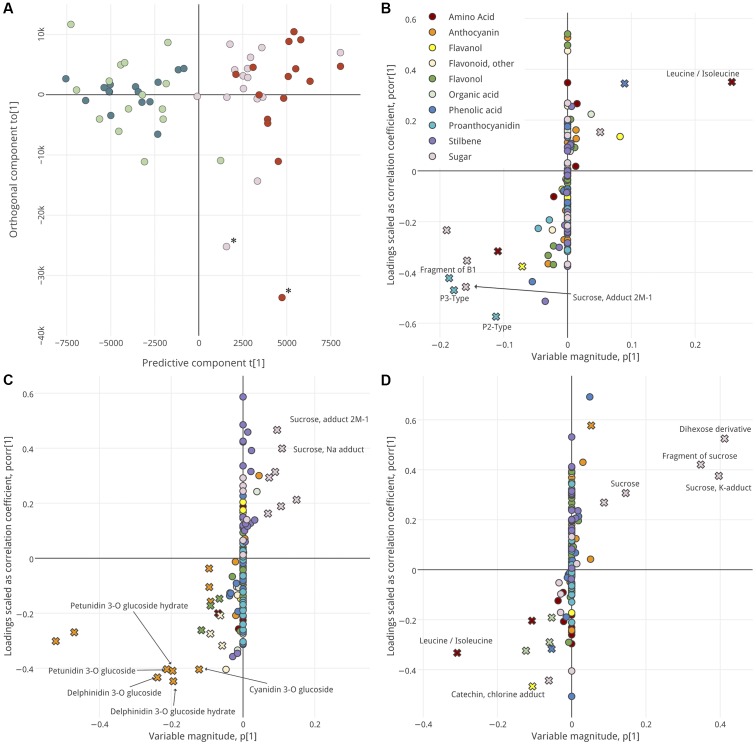
OPLS-DA analysis, by ripening group, of seed **(A,B)**, skin **(C)**, and pulp **(D)**. Score plot **(A)** shows separation of samples in analysis of seed. Hotelling’s T2 Ellipse (95%) not shown, but samples outside ellipse denoted with asterisk. Samples are distributed along a predictive component (*x*-axis) and orthogonal component (*y*-axis) and are colored per their ripening class: GH, green; GS, light green; PS, pink; RS, red. S-plots **(B–D)** show the influence of metabolites on sample segregation. Metabolites with high variable importance (VIP > 2) are indicated with a cross symbol. Putative biomarkers are labeled. Interactive versions of **(B–D)** are provided as in **Presentation 1**.

Prior to this, however, Principal Component Analysis (PCA) of all samples (including all annotated metabolites and unidentified features) revealed that the three berry tissues were remarkably distinct in their metabolite profiles (**Supplementary Figure S1**), with anthocyanins and stilbenes highest in and positively correlated with skin and with proanthocyanidins and flavanols in seed. Most flavonols and other flavonoids were associated with skin tissues. The distinct metabolic profiles of each tissue warranted analyzing each tissue separately to resolve any differences among the groups of interest. Interestingly, a high degree of similarity among the GH and GS berries and among the PS and RS berries was observed such that we were unable to model their differences with high predictability (Q2). This might indicate that the ripening classes are more similar to one another late in ripening than they were at véraison, but this is impossible to say conclusively without equivalent measures at véraison. The four ripening classes were aggregated into two groups which could be reliably well-modeled by OPLS-DA in each tissue (**Figure 2**)—Lagging (GH + GS) and Advanced (PS + RS). Still, the original ripening classes are colored in **Figure 2**.

**FIGURE 3:**
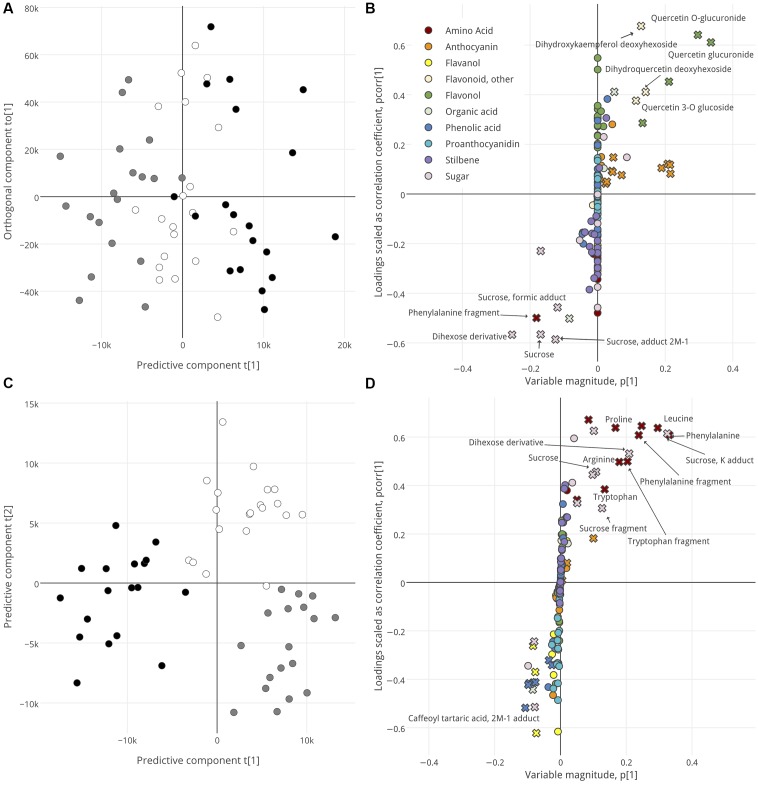
OPLS-DA analysis, by time sampled, of skin **(A,B)**, and pulp **(C,D)**. Score plots **(A,C)** show separation of samples. For skin, samples are distributed along a predictive component (*x*-axis) and orthogonal component (*y*-axis). For pulp, samples are distributed along the first two predictive components. Samples are colored per their collection date: t1, black; t2, white; t3, gray. S-plots **(B,D)** show the influence of metabolites on sample segregation. Metabolites with high variable importance (VIP > 2) are indicated with a cross symbol. Putative biomarkers are labeled. Interactive versions of B and D are provided in **Presentation 1**.

For each tissue, OPLS-DA was used to identify metabolites that define the ripening groups (**Figure 2** and **Supplementary Figure S2**) and define the intra-cluster metabolic changes during some of the latest stages of ripening (**Figure 3**). Model parameters are summarized in **Data Sheet 2**. Score plots (**Figure 2A** and **Supplementary Figure S2**) were used to visualize segregation among the samples, with the predictive component describing between-group differences and the orthogonal component describing within-group differences. For each tissue, the metabolic profiles of Lagging and Advanced berries were distinct, with no clear trends in within-group variance that could clearly be attributed to the original ripening classes (GH vs. GS and RS vs. PS). Next, S-plots with VIP integration were used to identify metabolites that best explain the segregation of ripening groups in each tissue (**Figures 2B–D**). S-plots show the covariance and correlation structure between the metabolites and predictive score. In other words, they show the reliability and influence of the metabolites on group segregation. The VIP score, also considered, is an additional metric that describes the extent to which any metabolite drives group distinctions. Metabolites with high VIP (>2) and relatively high |*p (corr)*| and |*p*| are putative biomarkers that define berries late in ripening that were Lagging or Advanced at véraison (**Figure 2**).

The seeds of Lagging and Advanced berries were defined by their high levels of proanthocyanidins and sugars versus leucine/isoleucine, respectively (**Figure 2B**). The skins of Lagging and Advanced berries were distinguished by high anthocyanins and sugars, respectively (**Figure 2C**), and the pulps of Lagging berries were high in and defined by leucine/isoleucine (**Figure 2D**), in contrast to the high levels of leucine/isoleucine found in Advanced berries’ seeds. Unsurprisingly, Advanced berry pulp was distinguishable by high levels of sugars (**Figure 2D**).

The score plots in **Figures 3A,C** also visualize segregation among the samples, but per their collection date and irrespective of their ripening class or group. No model that passed all acceptable thresholds upon cross-validation could be established to describe metabolic differences in seed or skin over time (seed, Q2 = 0.25, CV-ANOVA *p* > 0.05; skin, Q2 = 0.27, CV-ANOVA *p* = 0.022). Because the OPLS-DA skin-by-time model was valid, we have included it here despite low Q2. Metabolites with high scores in the corresponding S-plot (**Figures 3B,D**) indicate why the late ripening stages observed were distinctive. The skins of t1 and t3 berries were distinguished by their high levels of flavonols and other flavonoids versus phenylalanine and sugars, respectively (**Figure 3B**), and the pulp of t1 and t2/t3 berries could be segregated on the basis of high phenolic acids versus amino acids and sugars, respectively (**Figure 3D**).

**FIGURE 4:**
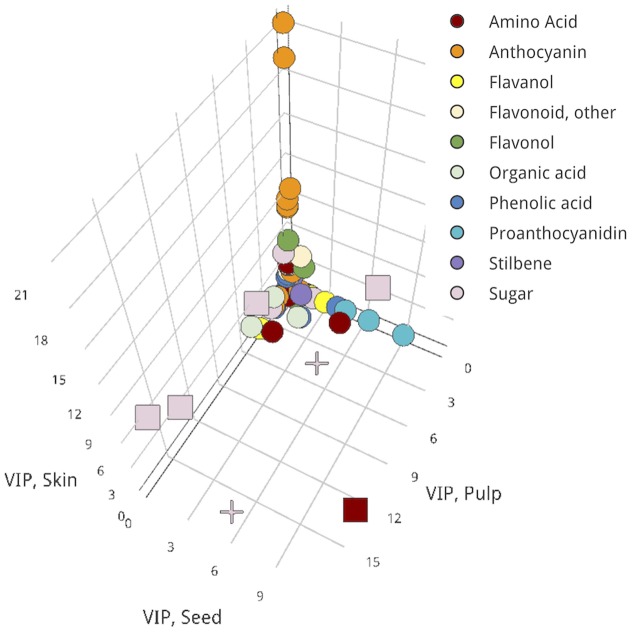
Three-dimensional VIP scatterplot for proposing in-common biomarkers across all three examined tissues. Pulp, *x*-axis; seed, *y*-axis; skin, *z*-axis. Metabolites for which a VIP score > 2 occurred in two tissues, square; in all tissues, cross; in only one tissue, circle. An interactive version of this figure is provided in **Presentation 1**.

Overall, biomarkers that define differences between ripening groups and were shared across all three tissues were exclusively sugars, specifically sucrose species and a di-hexose derivative. Leucine/isoleucine was an in-common biomarker between seed and pulp that distinguished Lagging from Advanced fruits (**Figure 4**).

Taken together, these results indicate that (1) variability late in ripening is associated with the developmental inequalities present at the ripening onset, (2) the metabolome remains dynamic post-harvest for pulp (and less for skin), with markers that define points during this late ripening period, and (3) that for seeds, the differences associated with ripening group at véraison exceeded those associated with change over the period observed.

### Trends in Intra-cluster Variance during Late Ripening

**FIGURE 5:**
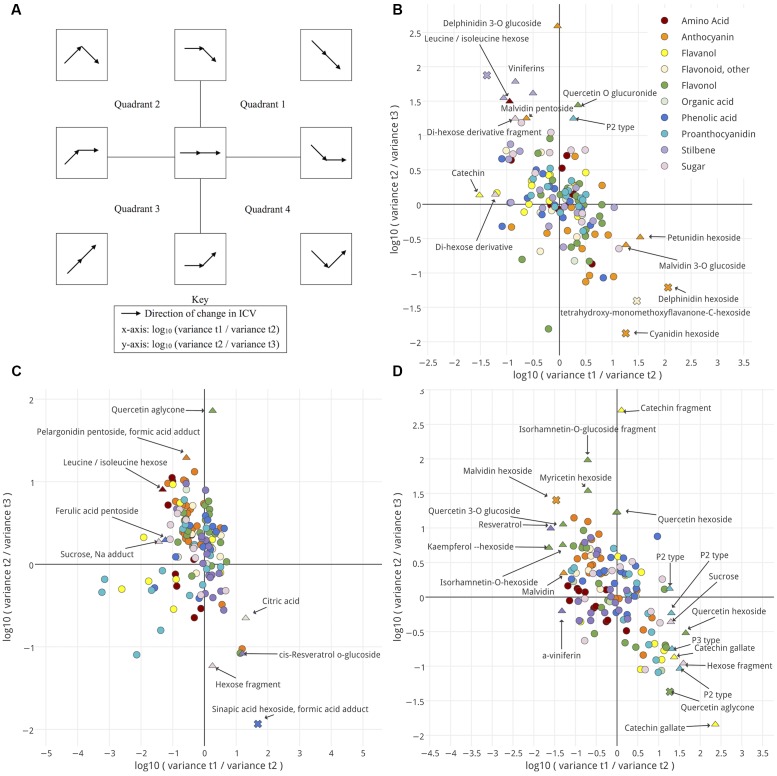
Changes in intra-cluster variance (ICV) for seed **(B)**, skin **(C)**, and pulp **(D)** between pairs of time points are shown. A schematic **(A)** illustrates how ICV changes over time for metabolites, depending on their location within **(B)** through **(D)**. Metabolites are colored by metabolite class. Shape indicates the outcome of an *F*-test for variance. No significant change between either pair of time points, circle; significant change between either pair of time points, triangle; significant change between both pairs of time points, cross. Significance threshold, *p*-value < 0.05. Interactive versions of **(B–D)** are provided in **Presentation 1**.

Next, trends in ICV late in ripening and the amount of ICV for metabolites with constant ICV were measured. The log_10_ fold-change in variance was plotted for each metabolite between pairs of sequential time points. This allows the visualization of ICV patterns for each annotated metabolite in the data (**Figure 5**). The ICV patterns characteristic of each region are summarized in **Figure 5A**. How metabolite variance behaved as fruits enter this late phase should provide evidence regarding whether the ripening classes were developmentally uniform or not. *Increasing ICV might suggest developmental inequality (Quadrants 2*, *3*, *and 4)*, *whereas decreasing ICV suggests migration toward developmental uniformity (*+*y/*+*x-axis and Quadrant 1). Metabolites with constant ICV would require further examination (Center).*

None of the annotated metabolites, in any tissue, significantly and exclusively increased or decreased in variance (**Figures 5B–D**). Though, several metabolites did demonstrate a significant change in ICV between a single pair of time points in each tissue. Further, some non-significant but observable trends appear upon examining classes of metabolites.

In seed (**Figure 5B**), amino acids, anthocyanins, flavonols, and organic acids predominantly localized on the right-hand side of the plot. Most metabolites that showed significant changes in ICV fell in quadrants 2 or 4. Several, however, experienced significant reductions in variation, characteristic of the +*y*-axis and +*x*-axis regions including several pigments, a P2-type proanthocyanidin, and quercetin-*o*-glucuronide. In skin (**Figure 5C**), leucine showed significant changes in ICV characteristic of quadrant 2 over the time-course and most amino acids fell in quadrants 2 and 3. Anthocyanins and sugars localized predominantly in quadrant 2, and phenolic acids, flavonols and other flavonoids in quadrants 1 and 2. Stilbenes mostly fell in quadrants 1 and 4, and proanthocyanidins into 2 and 3. Like seed, most metabolites in skin that showed significant changes in ICV fell in quadrants 2 or 4, and few metabolites showed no increase in ICV. In skin, this included quercetin aglycone, citric acid, and a glucoside of *cis*-resveratrol. For pulp (**Figure 5D**), stilbenes and amino acids occurred in quadrants 2 and 3, anthocyanins and other flavonoids predominantly in quadrant 2, phenolic acids in 1 and 2, and flavanols in 1 and 4. Like seed and skin, most metabolites in pulp that showed significant changes in ICV over the time course fell in quadrants 2 and 4, and several exhibited reductions in ICV either between t1 and t2 or t2 and t3. In pulp, these included sucrose and a hexose fragment, several proanthocyanidins, flavanols, and flavonols.

**FIGURE 6:**
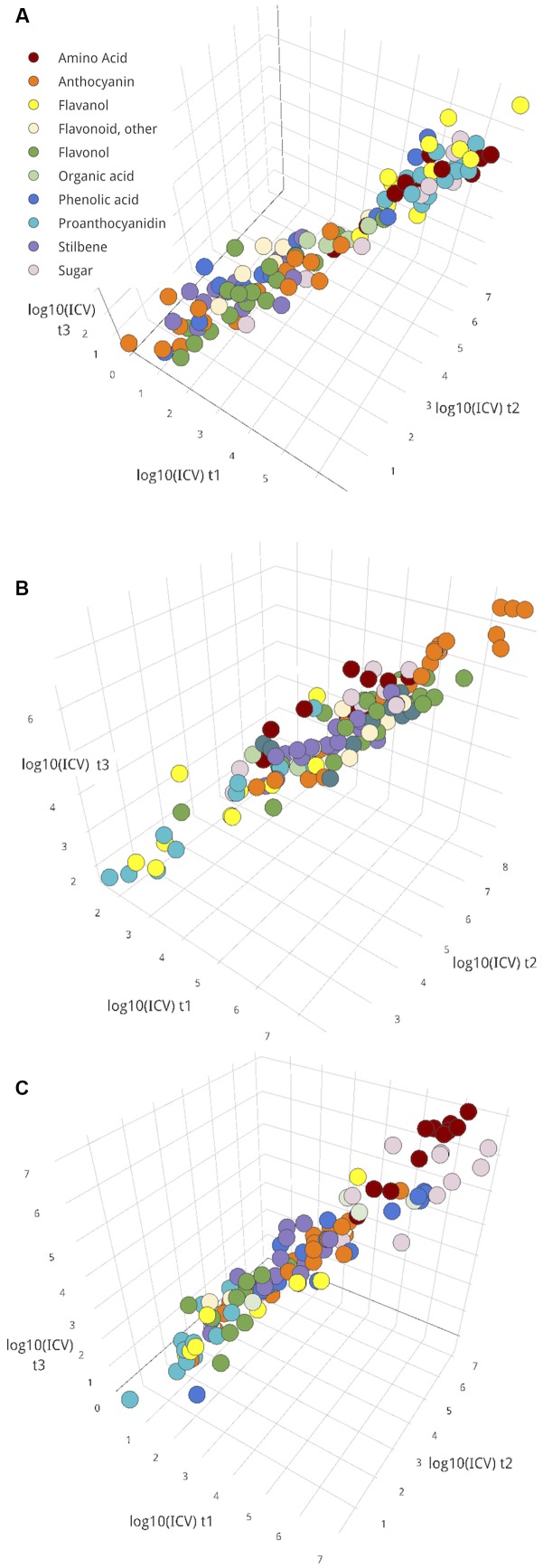
The log_10_ magnitude of variances for metabolites with constant variance (no significant change between pairs of time points) for seed **(A)**, skin **(B)**, and pulp **(C)** tissues. ICVs at t1, t2, and t3 are plotted on axes *x*, *y*, and *z*, respectively. For plot inclusion, *F*-test for variance, *p* **>** 0.05 between both pairs of time points for the metabolite. Interactive versions of these plots are provided in **Presentation 1**.

Overall, though, most metabolites did not show significant changes in ICV over the time-course (**Figures 5B–D**). Taking these metabolites, the magnitude of variance that persisted in the cluster was explored (**Figure 6**). This group could include metabolites with persistently high or low variance across the ripening classes throughout this study; in other words, the amount of difference between ripening classes did not significantly change over time for these metabolites, and that difference could be either large or small. The magnitude of ICV also provides insight into the developmental uniformity of berries in the cluster. *High*, *constant ICV might suggest persistent developmental differences*, *whereas low*, *constant ICV suggests developmental uniformity.*

Classes of metabolites tended to exhibit similar levels of ICV, so ICV was summarized per metabolite class in **Table 1**. In all tissues, amino acids had comparatively high levels of persistent ICV. Some of the most contextually important metabolites for each tissue tended to be among the most persistently variable– proanthocyanidins and flavanols in seed (**Figure 6A**), anthocyanins in skin (**Figure 6B**), and sugars in pulp (**Figure 6C**). In addition, flavonols and phenolic acids in seed (**Figure 6A**) and flavanols and proanthocyanidins in skin (**Figure 6B**) were among the least variable groups of metabolites.

**Table 1:** Tukey HSD tests comparing variances of metabolites classes within individual sampling dates.

	Tukey test comparison outcome			Mean log_10_(ICV) for metabolite class		
*Metabolite class*	*t1*	*t2*	*t3*	*t1*	*t2*	*t3*
**Seed**					
Proanthocyanidin	A	A	A	5.52	5.43	5.31
Amino acid	A	A	A	5.36	5.3	5.15
Flavanol	A	A	A	5.33	5.58	5.41
Sugar	A	AB	AB	5.03	5	4.53
Organic acid	AB	ABC	ABC	4.06	3.8	3.97
Flavonoid, other	BC	BC	BC	3.04	3.32	3.25
Phenolic acid	BC	C	C	2.94	3.14	3.13
Flavonol	BC	C	C	2.57	2.34	2.4
Anthocyanidin	BC	C	C	2.35	1.98	2.31
Stilbene	C	C	C	2.11	2.26	2.15
**Skin**					
Anthocyanidin	A	A	A	6.21	6.55	5.65
Flavonoid, other	A	AB	A	5.79	5.74	5.51
Flavonol	A	AB	A	5.77	5.83	5.44
Phenolic acid	A	AB	A	5.66	5.64	5.14
Sugar	A	AB	B	5.33	5.8	3.42
Stilbene	A	B	B	5.3	5.23	3.63
Amino acid	A	AB	A	5.17	5.85	6.07
Organic acid	AB	ABC	A	4.49	4.93	5.36
Flavanol	B	C	A	3.57	3.86	5.57
Proanthocyanidin	B	C	AB	3.4	3.59	4.6
**Pulp**					
Sugar	A	A	A	5.8	5.62	5.65
Amino acid	A	A	A	5.55	6.14	6.23
Organic acid	ABC	ABC	AB	4.44	4.37	4.59
Phenolic acid	B	B	B	4.21	4.2	3.92
Anthocyanidin	BCD	BC	BC	3.47	3.76	3.42
Stilbene	CD	BC	BC	3.1	3.44	3.35
Flavanol	BCD	CD	BCD	3.04	2.6	2.88
Flavonoid, other	DE	BC	BCD	2.66	3.18	2.82
Flavonol	DE	CD	CD	2.6	2.67	2.67
Proanthocyanidin	E	D	D	1.76	1.49	1.63

*In each metabolite class, only metabolites with persistent variance (as in **Figure 6**) were included in comparisons. Groups that do not share letters in common are significantly different, *p*-value < 0.05*.

These results indicate that the tendency toward reduced ICV is rare late in ripening, that variability will remain constant over this period for most metabolites (at least those considered here), and that high variability was often observed for the most spatio-contextually relevant metabolites.

## Discussion

This study predominantly examined the extent and behavior of intra-cluster variation late in the ripening phase and, similar to others who observed variation among berries, we observed variability between ripening classes (Carroll et al., 1978; Barbagallo et al., 2011; Kontoudakis et al., 2011; Rolle et al., 2011; Liu et al., 2016). In contrast to earlier studies which used berry density, color or weight classes to classify fruits and assess metabolite differences, we directly assessed variation associated with uneven ripening onset and, therefore, developmental inequality. If fruit uniformity at harvest is desirable, then understanding how metabolites accumulate in a developmentally diverse cluster of fruits, particularly late in ripening, should aid the identification of biomarkers to improve harvest decisions. We propose an approach to identify markers in the future and, given our data, the features that make this challenging. First, our inability to model four distinct ripening classes (instead, modeling Lagging vs. Advanced), which at véraison were distinguishable, does support some reduction in developmental variation across the classes as was observed in terms of gene expression and berry size by others (Gray and Coombe, 2009; Gouthu et al., 2014). However, development-associated differences among the ripening groups over the late-ripening period were still identifiable, and there were diverse patterns in ICV except a significant, continual reduction in variation.

Fruits within a cluster are strong, competitive sinks during ripening (Coombe, 1988; Davies et al., 1999). However, changes in sugar concentration are more associated with dehydration than sugar import into berries near commercial harvest (Coombe and McCarthy, 2000). This change delineates the majority of the ripening phase from that observed in this study. Fruits may indeed undergo a reduction of intra-cluster variation during ripening (Gray and Coombe, 2009; Gouthu et al., 2014), but if fruits were truly developmentally uniform or were approaching uniformity, spatio-contextually relevant metabolites would have low ICV or only have exhibited reductions in ICV as fruits enter this late stage. Instead, the re-divergence of the ripening classes in terms of °Brix and other metabolites, as well as persistently high variance in others indicates that the fruits remain developmentally distinct. Metabolic uniformity does not necessarily imply developmental uniformity. This contrasts the conclusions of Gray and Coombe (2009), wherein fruits must developmentally synchronize to proceed into subsequent growth stages. In the interest of improving harvest decisions, trends in ICV may be a worthy consideration, given that if one waits longer to harvest, for instance, there is no guarantee that variation will continually reduce.

In each of the tissues studied, amino acids were among the most variable overall. Most amino acids showed no significant changes in ICV over this experiment, except for leucine/isoleucine hexose in seed and skin. In addition, leucine/isoleucine was able to distinguish ripening groups in seed and pulp. Significant differences between ripening classes were observed at one or more time points for arginine, phenylalanine, leucine/isoleucine, leucine/isoleucine hexose, and tryptophan; these amino acids and proline were also able to distinguish t1 from t3 berry pulp (**Supplementary Figure S3**). Together, proline and arginine constitute 90% of the Nitrogen content in grape juice and influence the perception of acidity in wine (Gerós et al., 2012). Arginine and phenylalanine are both sources of Yeast Assimilable Nitrogen (YAN), and phenylalanine specifically is the precursor for the phenylpropanoid pathway giving rise to flavonoids and stilbenes. As the most abundant yeast-assimilable, N-containing metabolite in juice, arginine content is one factor in the production of fruity and floral wine aromas (Gutiérrez et al., 2015). Lagging berries had significantly higher levels of arginine and phenylalanine than pink and red berries at one or more times in and pulp tissues (**Supplementary Figure S3**). skin More specifically, in skin, the level of arginine in GH berries was significantly higher than in PS and RS berries at times 1 and 3; arginine was significantly higher in GS than PS and RS at t2. Likewise, the level of phenylalanine in skin was significantly higher in GS than in PS and RS at t2. In pulp, the level of phenylalanine was significantly higher for GH berries than the other classes at t2 and RS at t3. Similarly, the pulp levels of arginine in GH berries were higher than PS berries at t2 and RS berries at t3. Developmental differences in either arginine or phenylalanine could be important determinants of the differences we observed among downstream secondary metabolites. Leucine/isoleucine was among the metabolites best able to distinguish the seeds and pulp of less from more advanced berries. Phenylalanine and leucine participate in the production of higher alcohols during fermentation, namely 2-phenylethanol and isoamyl alcohol, the most abundant higher alcohols found in wine; these higher alcohols affect the aromas of wine and model solutions (Yoshizawa et al., 1961; Äyräpää, 1967; Vilanova et al., 2013; Noguerol-Pato et al., 2014; Vidal et al., 2014; Cameleyre et al., 2015). Previous work has implicated 2-phenylethanol in Pinot noir aroma, the variety used in this study (Miranda-Lopez et al., 1992; Girard et al., 2001). Finally, the potential implications of developmental inequalities in tryptophan are also interesting. Tryptophan is a precursor of auxin, a major regulator of fruit development and suspected precursor of 2-aminoacetophenone (AAP), an off-aroma described in white wines and the production of which can vary with harvest time (Hoenicke et al., 2001, 2002; Maeda and Dudareva, 2012; Schneider, 2014). Future studies could shed more light on how amino acid inequalities between individual berries originate and possibly propagate other metabolite inequalities, for better or worse.

Although ripening groups were distinguishable in each of the tissues examined, t1 vs. t3 fruits were only reliably differentiable in pulp and per their sugar and amino acid content. These observations add to previous reports which also demonstrate pulp continues to undergo metabolism and transcriptomic changes during dehydration on and off the vine (Bellincontro et al., 2004; Costantini et al., 2006; Moreno et al., 2008; Rolle et al., 2009; Toffali et al., 2011; Bowen and Reynolds, 2015; Khairallah et al., 2016; Lukić et al., 2016; Zenoni et al., 2016). For seed, the OPLS-DA and ICV patterns present seemingly contrasting results. OPLS-DA was unable to define seeds by their collection date, though was able to segregate ripening groups, and significant changes in ICV were observed for some metabolites between pairs of time points in seed. This suggests that the change in ICV over time in seed was not sufficiently large so as to define one time point versus another even though the difference between ripening groups may have expanded or contracted for some metabolites. Véraison for an individual berry marks the onset of ripening and coincides with the initiation of seed maturation, tannin oxidation, a cessation of seed growth, and seed dehydration (Ristic and Iland, 2005). The lack of or inability to observe changes is not entirely unexpected, given that the seed has matured by this period (Ristic and Iland, 2005) and changes that occur over time in seed might only be observed over longer time scales than in this study. Perhaps underlying factors that contributed to differences in seeds early in their development also influence seed composition after seeds have completed maturation such that they are distinguishable past the largest phases of seed development.

Tartaric and malic acids compose ∼90% of total berry acidity. Both acids are considered in harvesting decisions and impact final wine composition and perception. Typically, levels increase in the berry up to 4 weeks after anthesis and decline during ripening (Kliewer et al., 1967; Lamikanra et al., 1995). Consistent with this expectation, tartaric acid was consistently highest in and able to distinguish t1 versus t3 fruits in pulp. Furthermore, tartaric acid was significantly higher in GH fruits’ pulp than in GS, PS, and RS (**Supplementary Figure S4**); tartaric acid was probably an unsuitable marker of Lagging fruits (by OPLS-DA) because GS was indistinguishable from the red berries over the time course (**Supplementary Figure S4**). Although OPLS-DA analyses indicate that changes in organic acids late in ripening among berries have more to do with a dynamic pulp over time, this observation upon close inspection suggests that developmental differences do persist such that GH is perpetually laggard if defined by its tartaric acid content. Further examination could reveal how these inequalities in primary metabolites occur in the first place, and the extent to which they, plus environmental and physiological factors, contribute to wine quality and ICV among secondary metabolites.

Among the secondary metabolites are berry phenolics, which contribute to the color, astringency, and bitterness of wines. Because of their importance, there is substantial interest in characterizing phenolic composition in a way to better inform harvest times and anticipate wine composition (Cagnasso et al., 2008; Tian et al., 2009; Kontoudakis et al., 2011). Persistently variable ripening classes would include, then, fruits at different levels of phenolic maturity. The color of red wines is influenced by anthocyanins and other phenolics (co-pigmentation). Anthocyanins were highest in Lagging fruits’ skin, were able to distinguish Lagging from Advanced berries, and were among the most variable group of metabolites in skin tissues. Interestingly, developmental differences among the ripening classes were well-described by anthocyanins; being developmentally delayed and possibly having passed into the period of pigment decline (Hilbert et al., 2015), respectively, GH and RS fruits generally had lower anthocyanin levels, whereas the intermediate classes (GS and PS) tended to exhibit higher pigment levels (**Supplementary Figure S5**). Bautista-Ortín et al. (2006) showed anthocyanin extraction was improved in later-harvested fruits, even though they have lower pigment levels overall, to such a degree that slight over-maturation of fruit is perhaps desirable for determining harvest times (Bautista-Ortín et al., 2006); the developmental distance between the ripening classes may not only impact the abundance of anthocyanins, but their extractability as well.

Proanthocyanidins make significant contributions to wine bitterness and astringency in addition to their co-pigmentation activities. Kontoudakis et al. (2011) showed that under-ripe berries will differ from high density counterparts in their contribution of seed versus skin tannins, degree of polymerization, and resultant wine quality. Proanthocyanidins and flavanols were among the most variable metabolites in seeds, but only proanthocyanins were highest in and distinguished Lagging seeds (**Figure 2B** and **Supplementary Figure S6**). Per their seeds’ proanthocyanidin content, pink and red berries could be considered developmentally ahead of green fruits. Again, however, changes in the seeds over time were not sufficient to define time points over this period. This result is consistent with Ristic and Iland (2005), who showed tannin accumulation closely tied to fruit development and ripening, peaking at véraison and declining in seeds as they dry, mature, and brown. Curiously, catechin accumulation also peaks at véraison in seeds and skin and declines during ripening (Kennedy et al., 2000; Downey et al., 2003a; Ristic and Iland, 2005), though flavanols did not define ripening groups in seeds as did the proanthocyanidins. Furthermore, proanthocyanidins and flavanols were the least persistently variable in skin tissues; the same trends in abundance (green > red) were not observed in skin even though they are contextually relevant within that tissue.

Unlike anthocyanins and proanthocyanidins, hydroxycinnamic acids were neither distinctive features of a ripening group, nor at remarkable levels of ICV. Upon closer examination, no significant differences were observed among the ripening classes for caftaric or coutaric acids in pulp, though their abundance did change over time (**Supplementary Figure S4**). However, significant differences in gallic acid (a hydroxybenzoic acid derivative) were observed among the ripening classes’ seeds, with higher concentrations observed in pink and red fruits than in green fruits’ seeds. This result is somewhat consistent with Tian et al. (2009), who showed higher levels of gallic acid in must for later harvested fruits and is additional evidence that RS and PS remained “ahead” of green fruits late in ripening.

Unlike other metabolite classes which provide evidence that fruits are developmentally distinct, stilbenes did not define either of the ripening groups, though many stilbenes showed relatively high levels of ICV in skin and changed in ICV between at least one pair of time points in several tissues. High levels of stilbenes are found in berry skin, with variability between cultivars (Sun et al., 2006), can be induced to resist pathogens, and increase from véraison to harvest in Pinot noir (Gatto et al., 2008). Sources of variation besides developmental differences may contribute to the high ICV observed for stilbenes. Similarly, flavonols were not effective markers of developmental differences, but did distinguish t1 from t3 fruit overall, and were highly variable in a contextually relevant tissue—skin (Downey et al., 2003b).

If either uniformity or minimal heterogeneity is optimal, then when metabolites of interest are at a desired level and uniformity across the cluster would be pertinent to harvest decisions and identifying measurable biomarkers for this purpose would be valuable. This, of course, is complicated by developmental inequalities within the cluster that persist. An ideal marker would (1) have persistently low ICV such that any berry, regardless of developmental stage, could be used as a representative individual and (2) change over time so that a particular level of the marker might be associated with low ICV for a set of metabolites of interest. Although sugars are important in harvest decisions, those annotated here would be unsuitable markers by this definition. The sugars in our metabolite library were the best indicators for segregating ripening groups and several were indicative of their diversity, not of their uniformity. Identifying such a marker would at least require a longer time scale, more frequent sampling intervals than used in this study, thorough field testing, and would benefit from expanding grape-specific metabolite libraries (Aretz and Meierhofer, 2016). This study contributes to the growing body of work and interest in uneven ripening, its implications, and is pertinent to an ever-evolving paradigm in harvest decision-making. To better understand heterogeneity within the cluster and its impacts, future studies should continue to segregate variation associated with inherently unequal “starting positions” as well as other factors, such as microclimate and position in and of the cluster (Reshef et al., 2017).

## Author Contributions

All authors assisted in critical revision of this manuscript, approve of the version submitted for publication, and have agreed to be accountable for all aspects of the work, including ensuring that all questions regarding the accuracy or integrity of the work are appropriately investigated and resolved. This work was conceived by AV, FG, and LD. The experimental design and metabolite extraction were performed by AV and MC. The LC-MS instrumentation and metabolite identification were performed by MC and FG. The description of the LC-MS methodology was provided by MC. The statistical analyses were performed and manuscript was composed by AV.

## Conflict of Interest Statement

The authors declare that the research was conducted in the absence of any commercial or financial relationships that could be construed as a potential conflict of interest.

## Footnotes

1 http://mzmine.github.io/

2 http://www.massbank.jp

3 http://www.hmdb.ca

## Supplementary Material

FIGURE S1 PCA bi-plot of all samples [*p (corr)*] and associated loadings [metabolites, *t (corr)*], plotted using components 1 and 2.

FIGURE S2 Score plots from OPLS-DA analysis, by ripening group, of skin **(A)**, and pulp **(B)** which show separation of samples. Hotelling’s T2 Ellipse (95%) is not shown, but samples outside the ellipse are denoted with an asterisk. Samples are distributed along a predictive component (*x*-axis) and orthogonal component (*y*-axis) and are colored per their ripening class: GH, green; GS, light green; PS, pink; RS, red.

FIGURE S3 Selected amino acid dynamics over the time course. Tukey HSD tests were used to compare ripening classes at individual time points. Significant differences between the ripening classes at single time points are denoted with different letters, *p*-value < 0.05. For visual ease, the points may be shifted slightly to the right. However, there are only three time points (t1, t2, and t3) at which measurements were taken.

FIGURE S4 Selected organic and phenolic acid dynamics over the time course. Tukey HSD tests were used to compare ripening classes at individual time points. Significant differences between the ripening classes at single time points are denoted with different letters, *p*-value < 0.05. For visual ease, the points may be shifted slightly to the right. However, there are only three time points (t1, t2, and t3) at which measurements were taken.

FIGURE S5 Selected anthocyanins over the time course. Tukey HSD tests were used to compare ripening classes at individual time points. Significant differences between the ripening classes at single time points are denoted with different letters, *p*-value < 0.05. For visual ease, the points may be shifted slightly to the right. However, there are only three time points (t1, t2, and t3) at which measurements were taken.

FIGURE S6 Selected proanthocyanidins over the time course. Tukey HSD tests were used to compare ripening classes at individual time points. Significant differences between the ripening classes at single time points are denoted with different letters, *p*-value < 0.05. For visual ease, the points may be shifted slightly to the right. However, there are only three time points (t1, t2, and t3) at which measurements were taken.

DATA SHEET S1 Metabolite annotation.

DATA SHEET S2 SIMCA OPLS-DA model parameters and score plot axes details.

PRESENTATION S1 Interactive figures.
