# Supplementary figures and images for: Metabolite Profiling Reveals Developmental Inequalities in Pinot Noir Berry Tissues Late in Ripening
Source: Front Plant Sci. 2017 Jun 30;8:1108. doi: 10.3389/fpls.2017.01108 (PMC5491620; doi:10.3389/fpls.2017.01108)

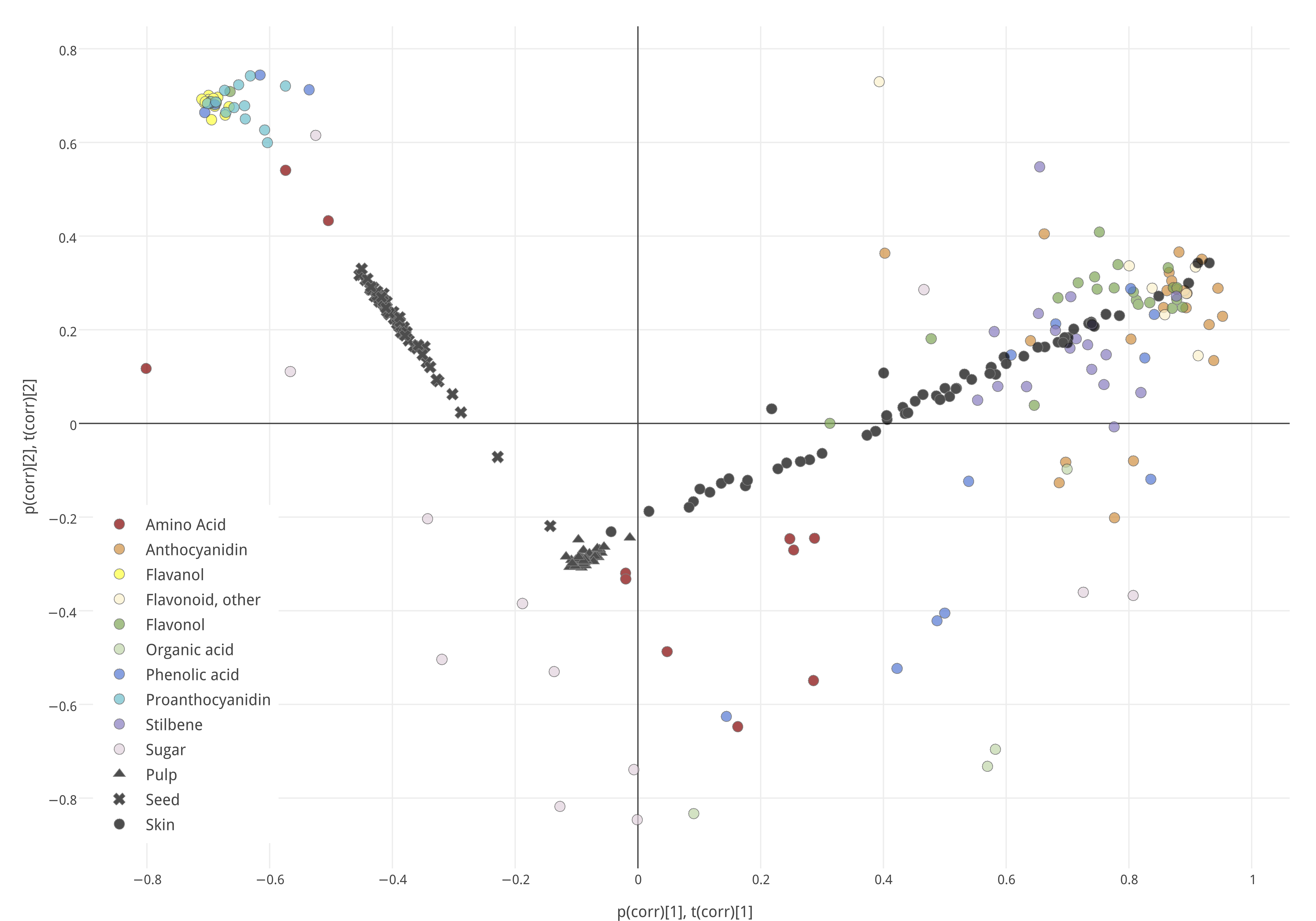

Supplement: FIGURE S1 — PCA bi-plot of all samples [p (corr)] and associated loadings [metabolites, t (corr)], plotted using components 1 and 2. [file Image_1.JPEG]

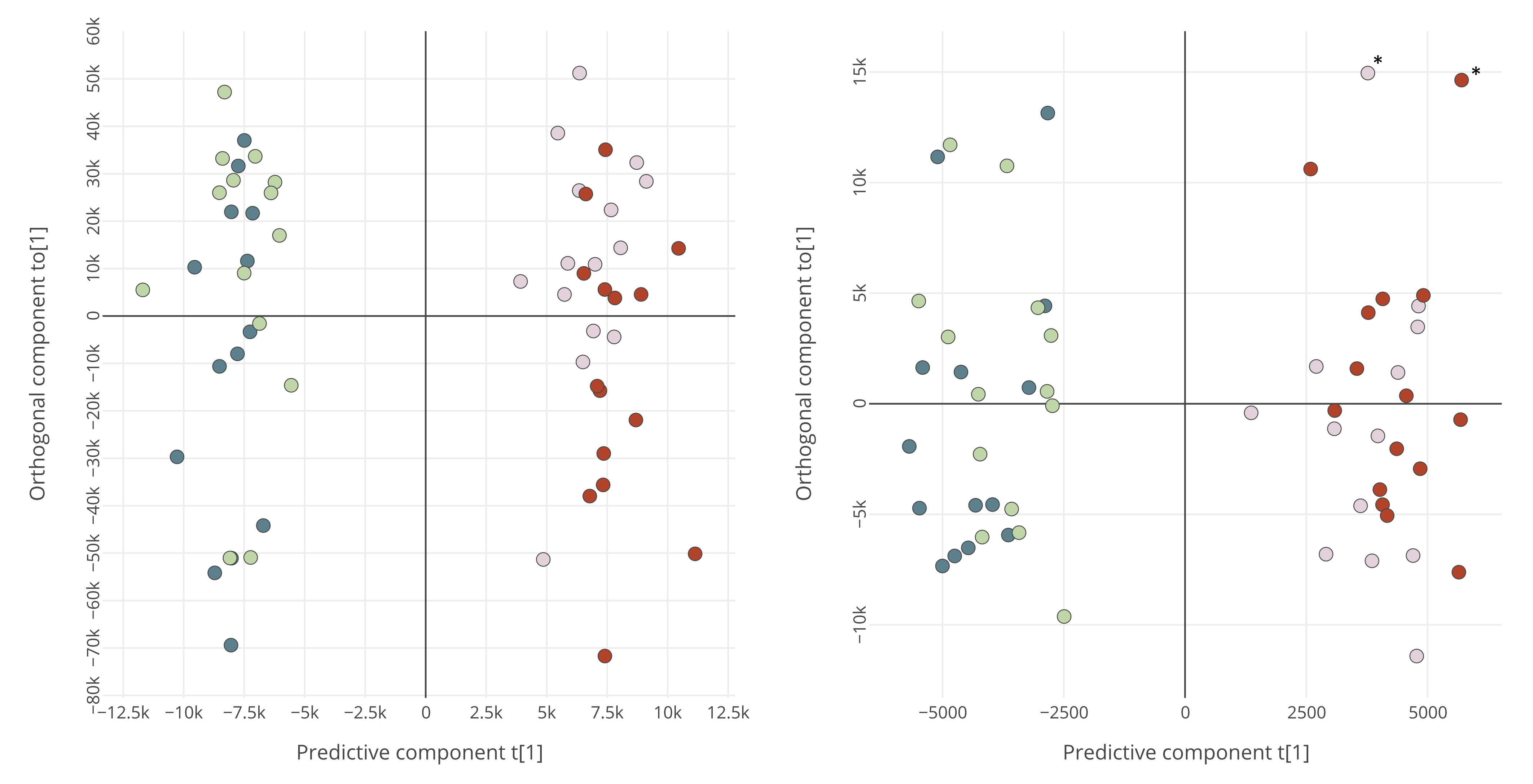

Supplement: FIGURE S2 — Score plots from OPLS-DA analysis, by ripening group, of skin (A), and pulp (B) which show separation of samples. Hotelling’s T2 Ellipse (95%) is not shown, but samples outside the ellipse are denoted with an asterisk. Samples are distributed along a predictive component (x-axis) and orthogonal component (y-axis) and are colored per their ripening class: GH, green; GS, light green; PS, pink; RS, red. [file Image_2.JPEG]

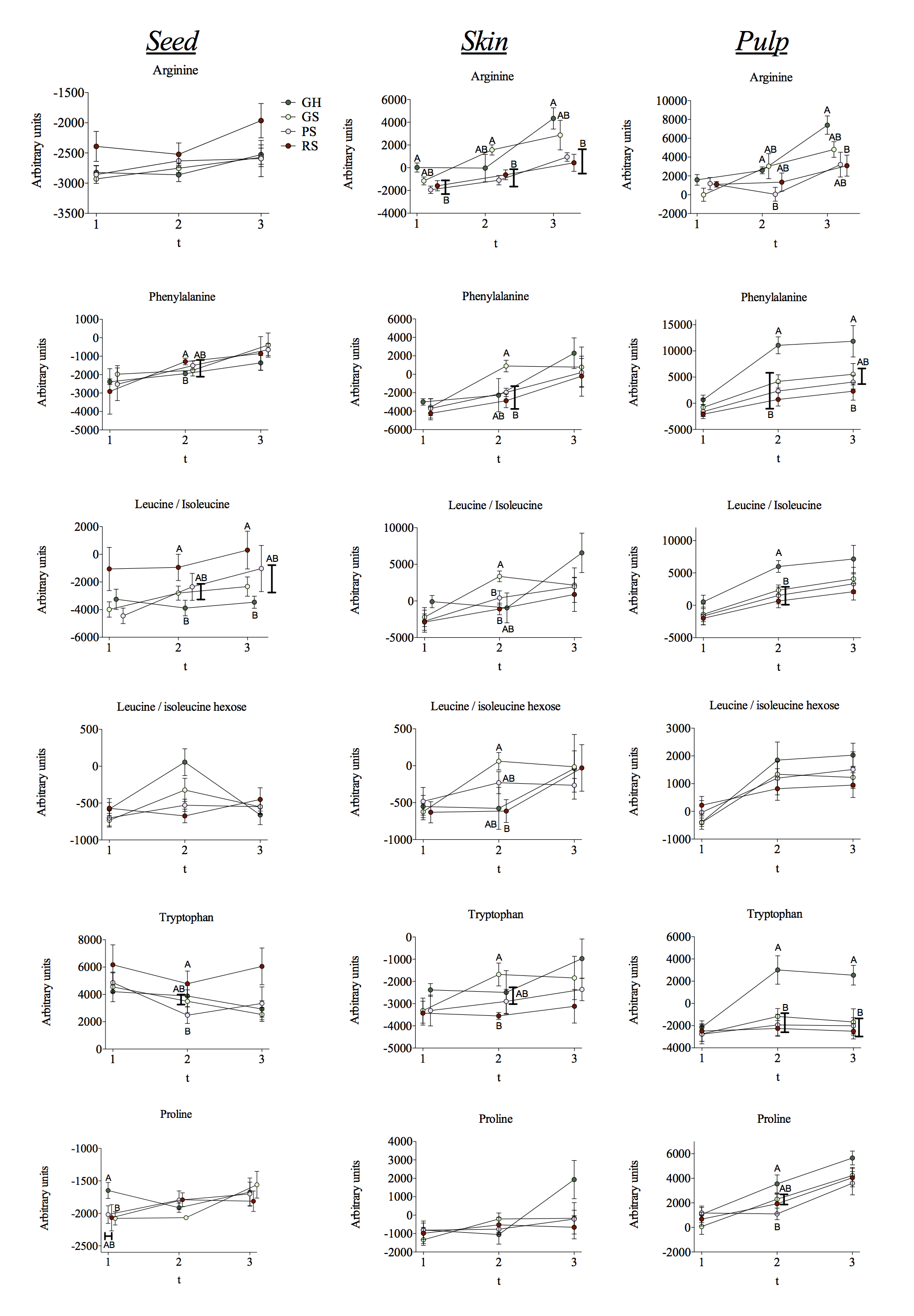

Supplement: FIGURE S3 — Selected amino acid dynamics over the time course. Tukey HSD tests were used to compare ripening classes at individual time points. Significant differences between the ripening classes at single time points are denoted with different letters, p-value < 0.05. For visual ease, the points may be shifted slightly to the right. However, there are only three time points (t1, t2, and t3) at which measurements were taken. [file Image_3.JPEG]

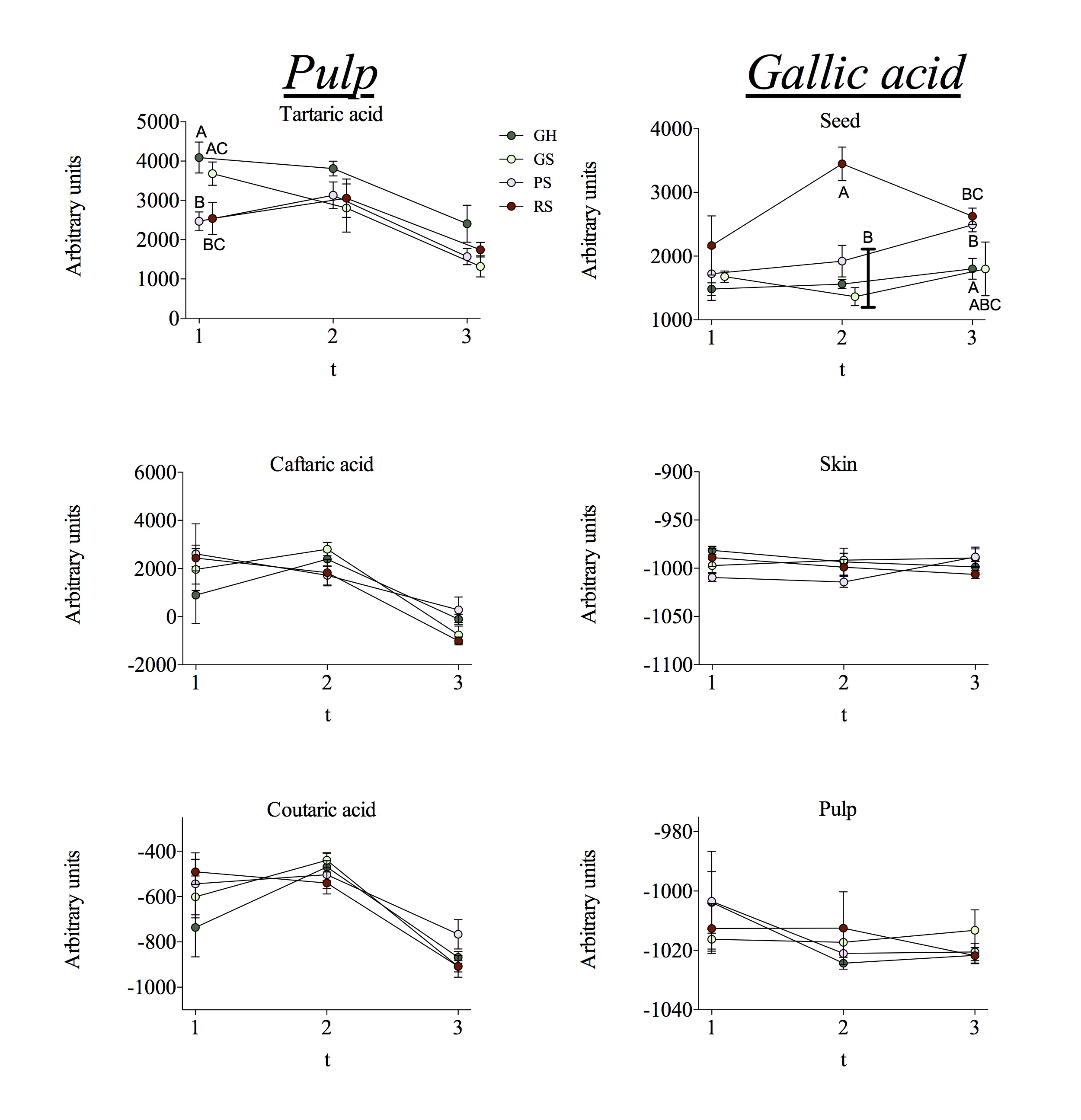

Supplement: FIGURE S4 — Selected organic and phenolic acid dynamics over the time course. Tukey HSD tests were used to compare ripening classes at individual time points. Significant differences between the ripening classes at single time points are denoted with different letters, p-value < 0.05. For visual ease, the points may be shifted slightly to the right. However, there are only three time points (t1, t2, and t3) at which measurements were taken. [file Image_4.JPEG]

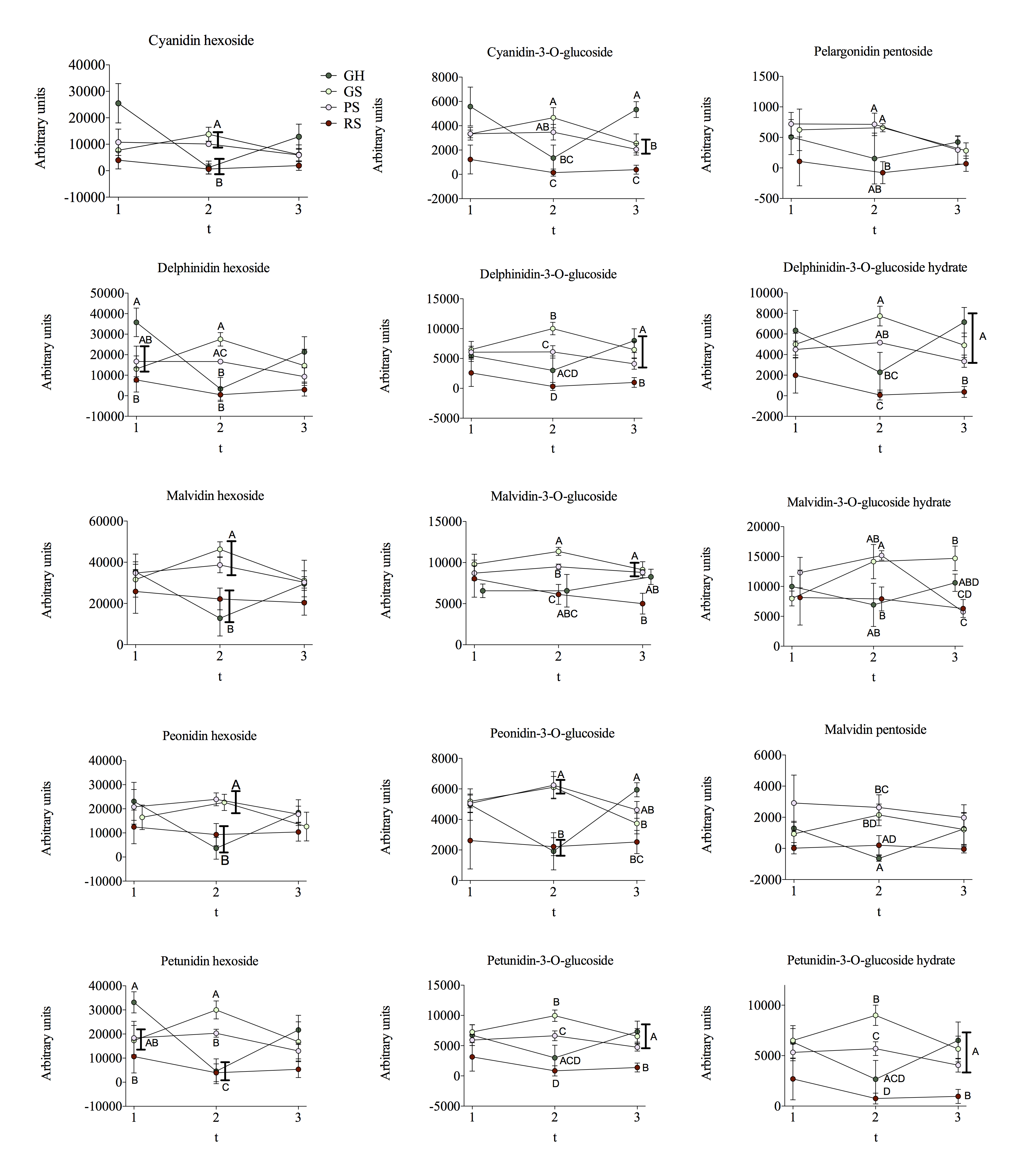

Supplement: FIGURE S5 — Selected anthocyanins over the time course. Tukey HSD tests were used to compare ripening classes at individual time points. Significant differences between the ripening classes at single time points are denoted with different letters, p-value < 0.05. For visual ease, the points may be shifted slightly to the right. However, there are only three time points (t1, t2, and t3) at which measurements were taken. [file Image_5.JPEG]

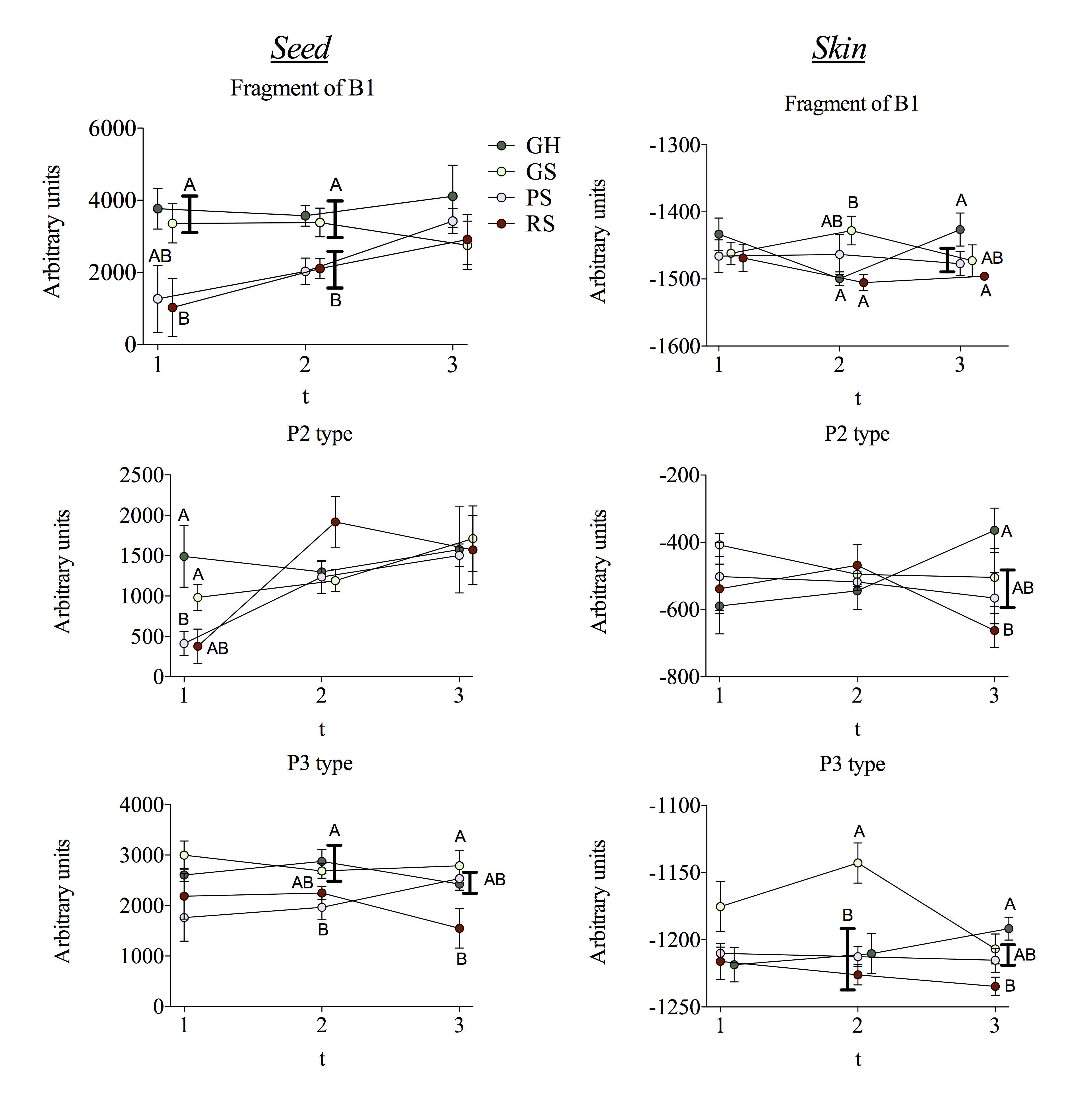

Supplement: FIGURE S6 — Selected proanthocyanidins over the time course. Tukey HSD tests were used to compare ripening classes at individual time points. Significant differences between the ripening classes at single time points are denoted with different letters, p-value < 0.05. For visual ease, the points may be shifted slightly to the right. However, there are only three time points (t1, t2, and t3) at which measurements were taken. [file Image_6.JPEG]
