# Supplementary material for: Metabolite Profiling Reveals Developmental Inequalities in Pinot Noir Berry Tissues Late in Ripening
Source: Front Plant Sci. 2017 Jun 30;8:1108. doi: 10.3389/fpls.2017.01108 (PMC5491620; doi:10.3389/fpls.2017.01108)
Supplement: DATA SHEET S2 — SIMCA OPLS-DA model parameters and score plot axes details. [file Data_Sheet_2.PDF]

M48: OPLS-DA Seed, Green vs. Advanced
x: 1.01478*t[1]
y: 1.19561*to[1]
R2X[1,RXSide Comp. 1]= 0.0845, 0.27
R2X(cum), R2Y(cum), Q2(cum) = 0.46, 0.798, 0.524

M50: OPLS-DA Skin, Green vs. Advanced
x: 1.00091*t[1]
y: 1.2065*to[1]
R2X[1,RXSide Comp. 1]= 0.0433, 0.7
R2X(cum), R2Y(cum), Q2(cum) = 0.9, 0.968, 0.838

M46: OPLS-DA Pulp, Green vs. Advanced
x: 1.00403*t[1]
y: 1.34925*to[1]
R2X[1,RXSide Comp. 1]= 0.0713, 0.157
R2X(cum), R2Y(cum), Q2(cum) = 0.486, 0.946, 0.635

M62: OPLS-DA Skin per time
x: 1.01722*t[1]
y: 1.12716*to[1]
R2X[1,RXSide Comp. 1]= 0.0411, 0.712
R2X(cum), R2Y(cum), Q2(cum) = 0.816, 0.368, 0.27

M57: OPLS-DA Pulp per time
x: 1.01951*t[1]
y: 1.05715*t[2]
R2X[1,2]= 0.124, 0.0567
R2X(cum), R2Y(cum), Q2(cum) = 0.47, 0.75, 0.55
